# MicroRNA-206 suppresses gastric cancer cell growth and metastasis

**DOI:** 10.1186/2045-3701-4-26

**Published:** 2014-05-05

**Authors:** Jun Ren, Hui-jie Huang, Yu Gong, Shen Yue, Li-ming Tang, Steven Y Cheng

**Affiliations:** 1 Department of General Surgery, Nanjing Medical University affiliated Changzhou No. 2 Hospital, 213000 Changzhou, Jiangsu, PR. China; 2 Department of Developmental Genetics, School of Basic Medical Sciences, Nanjing Medical University, 210029 Nanjing, Jiangsu, PR. China

**Keywords:** Gastric cancer, miR-206, Metastasis, Tumor suppressor

## Abstract

Gastric cancer is one of the leading causes of cancer death world-wide and carries a high rate of metastatic risk. In addition to other protein-coding oncogenes and tumor suppressor genes, microRNAs play an important role in gastric cancer tumorigenic progression. Here, we show that miR-206 is expressed at markedly low levels in a cohort of gastric tumors compared to their matching normal tissues, and in a number of gastric cancer cell lines. Down-regulation of miR-206 was particularly significant in tumors with lymphatic metastasis, local invasion, and advanced TNM staging. We find that forced expression of miR-206 suppressed the proliferation, colony-formation, and xenograft tumorigenesis of SCG-7901 cells, a line of gastric cancer cells. Forced expression of miR-206 also suppressed SCG-7901 cell migration and invasion, as well as metastasis in cell culture or tail-vein injected mouse models, respectively. The anti-metastatic effect of miR-206 is likely mediated by targeting metastasis regulatory genes STC2, HDAC4, KLF4, IGF1R, FRS2, SFRP1, BCL2, BDNF, and K-ras, which were drastically down-regulated by stable expression of exogenous miR-206 in SCG-7901 cells. Taken together, our results indicate that miR-206 is a tumor suppressor of gastric cancer acting at steps that regulate metastasis.

## Introduction

Gastric cancer (GC) ranks the fourth most common cancer and the second leading cause of cancer-related death worldwide [1]. The prognosis for patients with advanced gastric cancer is poor, despite recent improvements in gastriectomy, chemotherapy, and radiotherapy [2]. Since GC is a polygenic disease arising as the result of multiple gene dysregulations, elucidation of the regulatory network governing GC tumorigenesis is imperative for developing novel targeted therapies.

Tumorigenesis in the stomach is a multistep process that entails genetic and epigenetic alterations of protein-coding as well as non-coding proto-oncogenes and tumor-suppressor genes. Interactions among host, environmental and bacterial factors also influence the disease progress. Helicobacter *pylori* (HP) infection has been established as a major risk factor for GC; the two histological subtypes of gastric cancer, the intestinal and the diffuse subtypes, are both associated with HP infection, which contributes to more than 80% of cases [3], especially in antral/body gastric cancer [4]. Mechanistically, HP infection was found to cause a twofold increase in EGF and EGFR in gastric antral biopsy tissues, but its eradication reduces the levels of both factors to those of the controls [5]. HP infection in patients with chronic gastritis is also significantly associated with down-regulation of E-cadherin due to increased promoter methylation [6], whereas eradication HP significantly reduced the levels of MMP-9, a metalloproteinase positively associated with tumor invasion and metastasis [7], in the antrum and the corpus mucosa [8].

In addition to HP infection, there is a multitude of causes leading to aberrant gene expression in gastric cancer, and the mechanism of gastric cancer metastasis is very complex as well. The Ras-dependent MAPK signaling, which eventually leads to increased proliferation in gastric cancer and is mainly caused by aberrations of K-ras activation, is frequently associated with the intestinal-type gastric cancer [9]. Stanniocalcin 2 (STC2), a secreted glycoprotein with important functions in calcium and phosphate homeostasis, was reported to be expressed in high levels cancerous gastric tissues, lymph node metastasis, and venous invasion [10]. The 5-year survival rate was significantly lower in patients with STC2 expression compared to patients without STC2 expression [11]. BDNF (Brain-derived neutrophic factor) expression was reported to show significantly increased expression in gastric cancer tissue compared to adjacent normal mucosa, and high levels of BDNF at the invasive front were correlated to vessel invasion, lymph node metastasis, peritoneal dissemination, and poor prognosis in gastric cancer patients [12]. Despite the progress in identifying the above regulatory genes, the precise underlying mechanism causing GC metastasis still remains to be determined.

Dysregulations of microRNAs play very important roles in tumorigenesis. The role of microRNAs in gastric cancer tumorigenesis has been well documented. Here, we focus on miR-206, which along with its closely related paralogs miR-1, 133a, and miR-133b, plays very important roles in muscle differentiation. These four microRNAs are highly conserved in sequence and genomic organization, and are called myomiRs because they were found to be expressed at high levels in the muscles [13]. MiR-1-1/miR-133a-2, miR-1-2/miR-133a-1, and miR-206/miR-133b form clusters in three different chromosomal regions in the human genome 20q13.33, 18q11.2, and 6p12.2, respectively. However, these miRNAs can act as tumor suppressors in various human cancers [14]; for instance, miR-1 and miR-133a were found to be frequently down-regulated in bladder cancers, and suppress tumor growth by targeting TAGLN2 [15]. MiR-1 and miR-206 were shown to possess similar tumor-suppressor roles in rhabdomyosarcoma through blocking c-Met expression [16]. MiR-206 was also reported to act as a tumor-suppressor in breast cancer [17], and lung cancer [18]. We report here that miR-206 expression is inversely associated with lymphatic metastasis, local invasion, and the TNM malignancy staging of GC, and force expression of miR-206 suppresses GC cell growth, tumorigenesis, and metastasis.

## Materials and methods

### Tissue samples

Surgically resected human gastric cancer and adjacent non-tumor tissues were obtained from 35 patients admitted in the Department of Gastrointestinal Surgery, the Affiliated Hospital of Nanjing Medical University (Changzhou Second People’s Hospital). The project was approved by the Research Ethics Committee of Nanjing Medical University, and a written consent was obtained from each patient enrolled in this study. All tissue samples were processed for storage in liquid nitrogen and H&E staining for pathological analysis.

### Cell lines and cell culture

Human gastric cancer cell lines SGC-7901, BGC-823, AGS, non-malignant gastric cell line GES-1, and HEK293T cells were purchased from the Cell Resource Center, Shanghai Institute of Biochemistry and cell Biology, the Chinese Academy of Sciences. These cells were maintained in a humidity incubator at 37°C, 5%CO_2_ and SGC-7901, BGC-823, and GES-1 cells were cultured in RPMI1640 medium, HEK293T cells in DMEM, and AGS cells in F12K medium, respectively. All culture media were supplemented with 10% fetal bovine serum and antibiotics.

### RNA extraction and quantitative real-time PCR

Total RNA from gastric tissues or cell lines were extracted using Trizol reagent (TaKaRa) according to the manufacturer’s instructions. cDNA was synthesized with the PrimeScript RT reagent kit (TaKaRa). Quantitative RT-PCR was carried out with a SYBR Premix Ex Taq kit (TaKaRa) on a 7500 real time PCR system (ABI) and analyzed with the SDS analysis software package (version 2.0.1, Applied Biosystems). PCR primers were obtained from Invitrogen, and the reaction was 95°C, 10 min,followed by 40 cycles of 95°C 15 seconds and 60°C, 1 min. The U6 snRNA was used as an internal control. The results were presented as fold change, calculated using the 2^-△CT^ method, and a ratio of expression in the tumors relative to the normal tissues less than 1.0 was considered as low.

### Vector constructs

Pri-miR-206 was amplified from normal human genomic DNA by PCR using primers: 206-For 5′-ATAAGAATGCGGCCGCAGATGCGGGCTGCTTCTGGA-3′ and 206-Rev 5′-AGCTTTGTTTAAACCCTTGGTGAGGGAGTCATTTGC-3′. The PCR fragment was inserted into the MSCV-P2GM vector to generate P2GM-miR-206. The empty P2GM vector was used as the control.

### Cell transfection

Plasmid transfection of SGC-7901 cells were carried out using lipofectamine 2000 (invitrogen), and stable clones carrying P2GM-miR-206 or the empty vector were selected for puromycin resistance (10 μg/ml). MiR-206 and negative control mimics were obtained from Ribobio (Guangzhou, China) and transfection of mimics in SGC-7901 cells was done using Oligofectamine (Invitrogen). Briefly, SGC-7901 cells plated out 1 day earlier were transfected with 100 nm of miR-206 or control mimics at reaching 50% confluence. After 48 h, the cells were processed for further analysis.

### Cell proliferation assay

MTT assay was used to estimate the proliferative rate of SGC-7901 cells. Briefly, SGC-7901 cells were trypsinzed 2 days after transfection and reseeded at 2.5 × 10^3^ cells per well into 96-well plates. MTT (3-(4, 5-dimethylthiazol-2-yl)-2, 5-diphenyl-tetrazoliumbromide) was added to the culture medium at specified intervals for xx hrs, and the absorbance at 490 nm was measured with a spectrophotometer. Each assay was performed in triplicates and repeated three times independently.

### Colony formation assay

Stable SCG-7901 cells carrying miR-206 or the empty control were trypsinized and replated at 1 × 10^3^ per well in 6-well plates. After 14 days in culture in RPMI1640 supplemented with 10% FBS, colonies were fixed with 3.7% methanol and stained with 0.1% crystal violet. Colonies containing at least 50 cells were scored. Each assay was performed in triplicates.

### Flow cytometry

SGC-7901 cells transfected with miR-206 mimic or negative control were trypsinized and resuspended in 1× binding buffer at 1 × 10^6^ cells/ml. 100 μl of this cell suspension was incubated with 5 l of FITC-Annexin V and 5 μl propridium iodide (PI) for 15 minutes in the dark. The reaction was terminated with the addition of 400 μl 1× binding buffer and analyzed with (FACSCalibur using the CellQuest software (Becton Dickinson). FITC-Annexin V-positive and PI-negative cells were considered as apoptotic and the experiments were carried out in triplicates.

### Migration and invasion assays

Wound healing cell migration was assessed by measuring the movement of cells into a acellular area created by scraping the confluent cell lawn with a 200 μl pipette tip. The wound closure was photographed 48 hr later under a microscope. For transwell migration assays, 5 × 10^4^ cells were added into the upper chamber of the insert (BD Science, Sparks, MD, USA), whereas 1 × 10^5^ cells were used for matrigel invasion assays. In these experiments, the cells were trypsinized and resuspended in serum-free medium before being seeded to the upper chamber. The full culture medium containing 10% FBS was added in the lower chamber. The cells were incubated in a humidified incubator at 37°C for 24 h. The cells on the membrane were fixed, stained, and counted. Each experiment was performed in triplicates.

### Xenograft tumor model

Young female athymic nude mice (6 weeks old) were purchased from the Model Animal Research Center of Nanjing University. Approximately 1.5 × 10^6^ stable SGC-7901 cells carrying P2GM-miR-206 or the empty vector were injected subcutaneously into the lower flanks of 5 nude mice. Tumor volumes were measured every 3 days from the sixth day post injection onward for 24 days before the animals were sacrificed. The final volume and weight were measured after the tumors were dissected. Nude mice were manipulated and cared for according to NIH Animal Care and Use Committee guidelines in the Experiment Animal Center of the Nanjing Medical University (Nanjing, Jiangsu, Province, P.R. China).

### Statistical analysis

Statistical analysis was performed using the SPSS software package (SPSS Standard version 16.0, SPSS Inc). Data were shown as mean ± SEM. The paired-samples *t*-test was used in the analysis of differential miR-206 expression between tumor and normal tissues. For in vitro and in vivo experiments, independent-samples *t*-test was used for assessing the significance of difference between the treatment and control groups. P value < 0.05 was considered as statistically significant.

## Results

### Down-regulation of miR-206 correlates with human gastric cancer tumorigenesis, invasion, and metastasis

**Figure 1:**
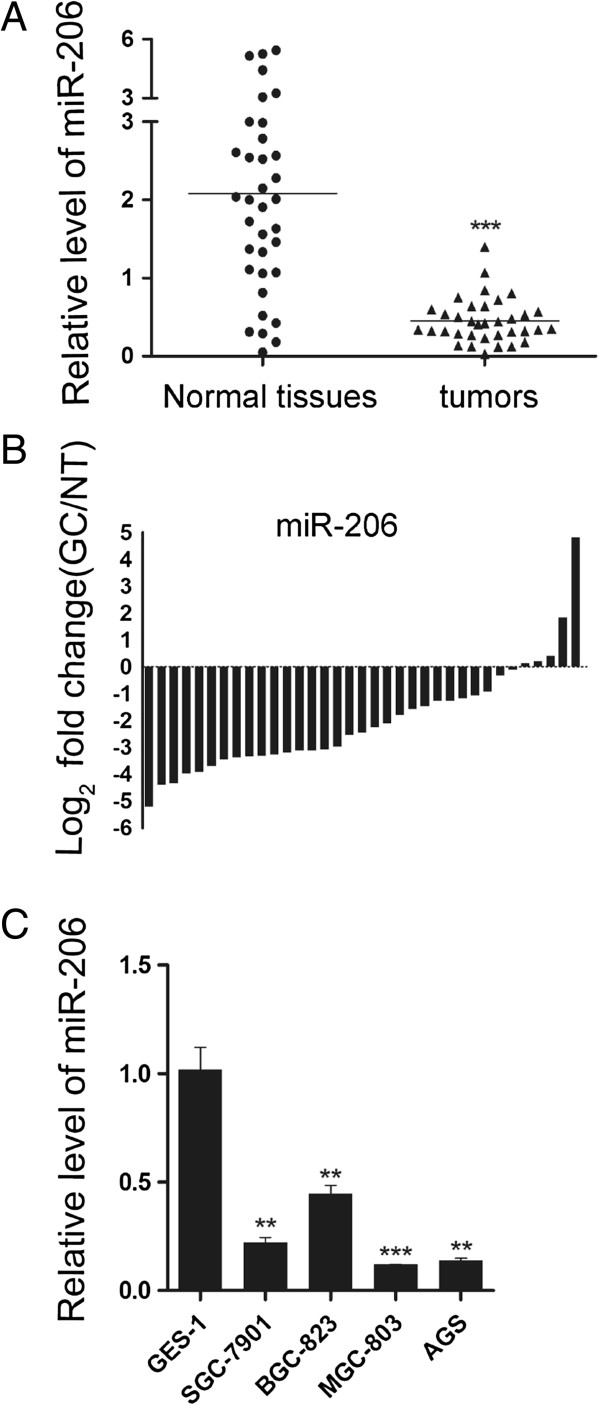
**Reduced levels of miR-206 expression in gastric cancer tissues and cell lines. (A)** Distribution of miR-206 expression in a cohort of 35 human GC and noncancerous tissues by qRT-PCR. The endogenous U6 RNA was used as the internal control. **(B)** Pairwise comparison of miR-206 expression between GC and matching non-cancerous tissues showing miR-206 expression was reduced in 86% (30/35) of the sample pairs. **(C)** Relative expression of miR-206 in four GC cell lines and a normal gastric cell line (GES-1).

In a genome wide survey for microRNA expression, we previously identified several members of the myomiR family, namely miR-1/133a and miR-133b/206, whose levels were reduced in a neuroectodermic cancer (unpublished results). These microRNAs were primarily known to function in myogenic differentiation and, to a lesser degree, the tumorigenesis of pediatric cancers. To explore if these myomiRs might also have a role in the tumorigensis of common cancers in the adult, we examined their expression levels by real-time stem-loop RT PCR quantification in a cohort of freshly resected gastric tumors and their matching normal tissues, and found that the median ratio of miR-206 relative to U6 snRNA of the 35 tumors was 4-fold lower than that of the normal tissues (Figure 1A). Pair-wise comparison indicated that over 85% of tumors showed greater than 2-fold reduction of miR-206 expression compared to their matching controls, with only two pairs showing increase (Figure 1B). Expression of miR-206 was also decreased in a number of human gastric cancer cell lines, including SGC-7901, BGC-823, MGC-803, and AGS cells, compared to that in GES-1 normal gastric cells (Figure 1C). Clinicopathological records of these tumors showed that the reduction of miR-206 expression was significantly associated with lymphatic metastasis, local invasion, and the TNM malignancy staging, but not with age, gender, tumor size or location (Table 1). These data imply that miR-206 may normally exert an inhibitory control on gastric tumorigenesis, invasion, and metastasis.

**Table 1:** Clinicopathologic characteristics of miR-206

**Categories**	**NO. of patients**	**Relative levels of miR-206 (mean ± SEM)**	**P-value**
Gender			
Male	21	0.49 ± 0.07	0.32
Female	14	0.40 ± 0.06	
Age (years)			
≥60	19	0.45 ± 0.05	0.93
<60	16	0.46 ± 0.09	
Diameter (cm)			
≥5	13	0.46 ± 0.09	0.90
<5	22	0.45 ± 0.05	
Location			
Middle and proximal third	23	0.47 ± 0.06	0.65
Distal third	12	0.42 ± 0.07	
Degree of differentiation			
well and moderately	12	0.48 ± 0.07	0.70
Poorly	23	0.44 ± 0.07	
Local invasion			
T1 + T2	10	0.65 ± 0.07	0.01
T3 + T4	25	0.37 ± 0.05	
Lymph node metastasis			
NO	14	0.57 ± 0.06	0.04
YES	21	0.38 ± 0.07	
TNM stage			
I + II	12	0.60 ± 0.07	0.02
III + IV	23	0.38 ± 0.06	

Expression of miR-133a was also found to be down-regulated in the tumors by the above criteria (Additional file 1: Figure S1), but expression of miR-1, whose genes are clustered with those encoding miR-133a [14], was not (Additional file 2: Figure S2). Since miR-206 is encoded by a unique gene, it was chosen for further analysis.

### MiR-206 inhibits gastric cancer cell growth

**Figure 2:**
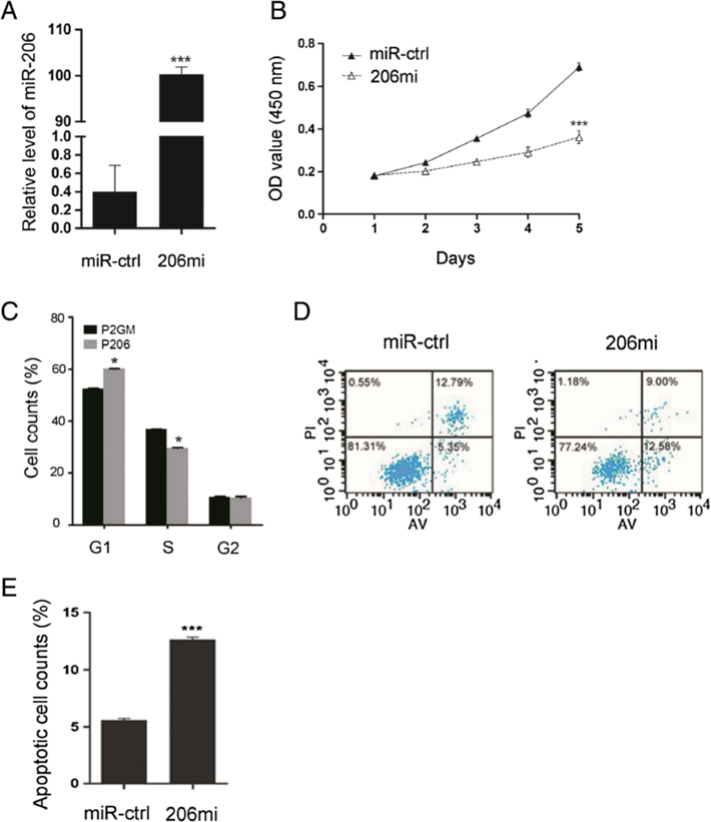
**Forced expression of miR-206 suppresses the proliferation of SGC-7901 gastric cancer cells. (A)** Total levels of miR-206 in SGC-7901 cells transfected with the miR-ctrl or the synthetic mimics of miR-206, 206mi (***P < 0.0001). **(B)** MTT assays for SGC-7901 cells transfected with miR-206 mimics at a final concentration of 100 nM. Starting from 24 h post-transfection, the assays was read every 24 h for 5 consecutive days. Results are expressed as means ± SEM from three independent experiments. (***P < 0.0001). **(C)** A histogram depicting cell cycle distribution of SGC-7901 cells transiently transfected with P2GM-miR-206 or P2GM-ctrl (100 nM). The results represent the means ± SEM of three independent experiments (*P < 0.05). **(D)** Cells staining positive for Annexin V-FITC and negative for PI at 48 h post-transfection were considered to have undergone apoptosis. **(E)** Average apoptotic rate of three independent experiments ± SEM are shown (***P < 0.0001).

To determine if miR-206 has the propensity to suppress gastric cancer tumorigenesis, we introduced a synthetic double stranded miR-206 mimics, 206mi, to alter the level of total miR-206 in SGC-7901 gastric cancer cells, which express a low level of miR-206 relative to that of GSE-1 normal control cells (Figure 1C), and monitored the rate of cell growth using the tetrazolium dye, 3-(4,5-dimethylthiazol-2-yl)-2,5-diphenyl tetrazolium bromide (MTT). Stem-loop PCR confirmed the elevated level of miR-206 in the transfected SGC-7901 cells (Figure 2A), and results from the MTT assay indicated that the growth rate of these cells was reduced markedly compared to that of the cells receiving non-specific control mimics, miR-ctrl, over a 5-day period (Figure 2B). We also generated a pre-miR-206 expressing plasmid in the P2GM vector, and fluorescence-activated-cell-sorting (FACs) analysis showed the prolonged G1-phase and the shortened S-phase in P2GM-206 (P206) transfected compared to the P2GM vector transfected SGC-7901 cells (Figure 2C), thus corroborating the result from the above 206mi mimic experiment. Artificially increasing the total level of miR-206 with 206 mi also resulted in a significant increase of apoptosis in 48 hrs in SGC-7901 cells over the control mimics transfected cells as determined by flow cytometric analysis of PI and Annexin V double uptake (Figure 2D and E). These data indicate that miR-206 is a potent anti-proliferative regulator of cultured gastric cancer cells.

### MiR-206 inhibits anchorage-dependent gastric cancer cell growth and xenograft tumor formation

**Figure 3:**
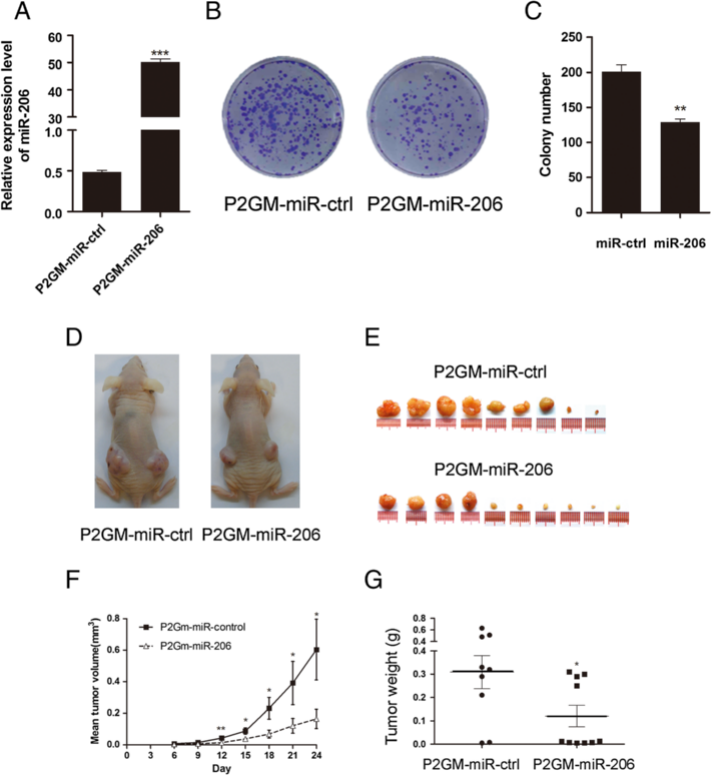
**MiR-206 suppresses anchorage-dependent GC cell growth and xenograft tumor formation. (A)** The expression level of miR-206 in P2GM-miR-206 stable cell line and vector-carrying control stable cells (***P < 0.0001)**. (B)** One thousand stable SGC-7901 cells of miR-206 and negative control were plated onto 6-well plates and a colony formation assay carried out. Representative results of colony formation of P2GM-miR-control, P2GM-miR-206. **(C)** The number of colonies was counted on the 14th day after seeding. Stable SGC-7901 cells of miR-206 grew to a lower density compared to NC. The results represent the means ± SEM of three independent experiments (**P < 0.01). **(D)** SGC-7901 cells with stable expression of miR-206 or not were inoculated subcutaneously into both flanks of nude mice(n = 5 per group). Representative photographs of nude mice 24 days after inoculation are shown. **(E)** The mice were killed and the tumors were weighted 24 days after inoculation, the xenografts with miR-206 overexpression were significantly smaller than the control xenografts. **(F)** shows tumor growth curve (*P < 0.05 and **P < 0.01). **(G)** Tumors from each group were weighed immediately after removal. The average tumor weight is indicated as means ± SEM (*P < 0.05).

To examine the long term impact of miR-206 on cell growth and colony formation, we generated a stable line of SGC-7901 cells, P2GM-miR-206, that expresses miR-206 from a constitutively active vector, MSCV-P2GM, and a line of empty vector-carrying cells, P2GM-miRctrl. First, we examined the expression level of miR-206 in these two stable cell lines and found that miR-206 was dramatically elevated in P2GM-miR-206 stable cell line (Figure 3A). Then we analyzed the effect of miR-206 on anchorage-dependent growth by sparsely seeding trypsinized cells directly onto petri dish and visualize the cell colonies by crystal violet staining following 14 days of culture. The results showed that the vector-carrying control stable cells formed significantly more colonies than the miR-206-expressing cells (Figure 3B and C). We further injected these stable cells subcutaneously into two bilateral lower backs of nude mice, and monitored tumor growth daily. Twenty-four days following the injection, these mice were sacrificed and the tumors were excised and weighed (Figure 3D and E). The results showed that the average volumes of miR-206-overexpressing tumors grew at a much slower pace than the control tumors and the mean final weight was significantly less than that of the controls (Figure 3F and G).

### MiR-206 inhibits gastric cancer cell invasion and migration

**Figure 4:**
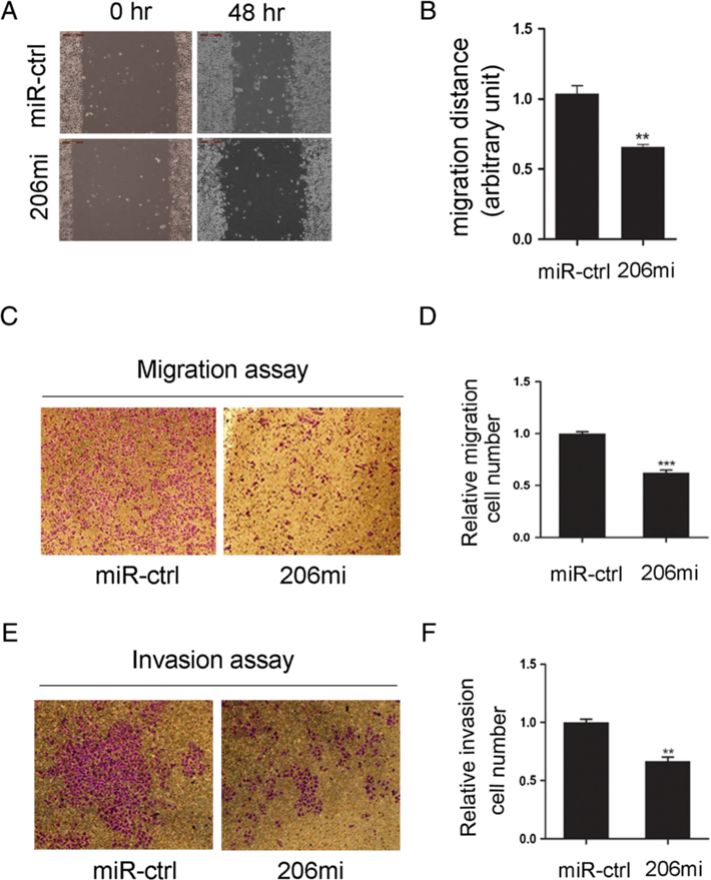
**MiR-206 suppresses GC cell migration and invasion in vitro. (A)** The wound-healing assay showed different cell motilities in miR-control, miR-206 cells. The ectopic expression of miR-206 obviously inhibited the migration of SGC-7901 cells. **(B)** shows the statistical results. Data represent the means ± SEM of three independent experiments. (**P < 0.01). Over-expression of miR-206 significantly impeded abilities of cell migration **(C)** and invasion **(E)** in miR-206-expressing stable SGC-7901 cells compared with negative control. **(D, F)** shows the statistical results respectively. Data represent the means ± SEM of three independent experiments. (**P < 0.01, ***P < 0.0001).

Reduced expression of miR-206 in locally invasive and metastatic tumors (Table 1) suggests that this microRNA may regulate cell migratory process. To determine if this indeed is the case, we carried out wound-healing and transwell migration assays using the miR-206-expressing stable SGC-7901 cells. The results showed that ectopic overexpression of miR-206 caused a suppression of cell migration in SGC-7901 cells as evident in the wound-healing assay (Figure 4A and B) and the transwell cell migration assay (Figure 4C and D). Ectopic overexpression of miR-206 further inhibited SGC-7901 cell invasion as demonstrated by the Matrigel invasion assay (Figure 4E and F). Hence, miR-206 displayed a suppressive property in cancer cell migration and invasion assays in vitro.

### miR-206 inhibits liver metastasis of GC in vivo

**Figure 5:**
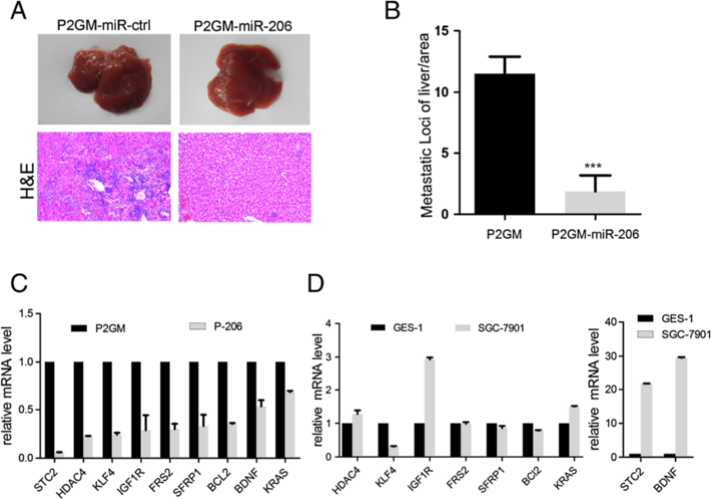
**MiR-206 inhibits liver metastasis of GC in vivo. (A)** Representative macroscopic pictures of mouse liver, 42 Days after inoculation, (The upper). Representative photographs of H&E stained spontaneous liver metastases. Magnification:10× (The lower). The results show that tumor nodules in P2GM-miR-206 group were smaller and fewer as compared to the control group. **(B)** Numbers of liver metastatic loci were counted and compared between mice bearing SGC-7901 vector control cells and SGC-7901 cells expressing miR-206. Data represent the means ± SEM, (***P < 0.0001). **(C)** Relative mRNA level of putative targets of miR-206 between SGC-7901-miR-P2GM and SGC-7901-miR-206 cells. **(D)** The relative mRNA level of putative targets of miR-206 between GES-1 and SGC-7901 cell lines.

To investigate the effect of miR-206 on metastasis in vivo, we carried out tail-vein injection of P2GM-miR-206 and the vector alone P2GM cells with nude mice, 42 days after injection, we harvested and photographed the livers (Figure 5A, upper panels). H&E staining of the tissue sections indicated a 7-fold lower number of metastatic tumor nodules per unit area in the livers injected with P2GM-miR-206 cells than those injected with P2GM control cells (Figure 5A, lower panels and B). Moreover, the sizes of tumor nodules in the P2GM-miR-206 cell-injected livers were much smaller than those of the control group (Figure 5A). This result strongly argues that miR-206 normally may have a tumor suppressor role preventing metastasis of gastric cancer cells.

To determine the mechanism by which miR-206 exerts its anti-metastatic role, we searched for possible miR-206 target genes using a combination of online microRNA target acquisition tools, and cross-referenced the resulting candidates to those that have been reported to play a role in metastasis. In a pool of 20 of such genes, we found 9 of them (Additional file 3: Table S1), namely STC2, HDAC4, KLF4, IGF1R, FRS2, SFRP1, BCL2, BDNF, and K-ras, were drastically down-regulated by miR-206 in the P2GM-miR-206 stable cells compared to their respective levels in the P2GM vector stable cells (Figure 5C). Some of these miR-206 target genes were markedly up-regulated in the parental SCG-7901 GC tumor cells compared to normal gastric GES-1 cells (Figure 5D), suggesting a likely causal event. Thus, miR-206 likely inhibits metastasis of GC tumor cells through a network of regulatory genes.

## Discussion

Although dysregulation of miRNAs was reported in various types of human cancers [19], aberrant expression and potential role of miRNAs in gastric cancers were understudied. Here, we demonstrate that miR-206 is frequently downregulated in human gastric cancers and cancer cell lines. In light of the reduced miR-206 expression reported in several types of tumors, our results suggest that reducing miR-206 expression is likely a prerequisite event in tumorigenesis. This requirement was made even more relevant when we found that low levels of miR-206 were significantly associated with lymphatic metastasis, local invasion, and TNM stage. This notion was corroborated by the outcomes of ectopic expression of miR-206 in GC cancer cell line SCG-7901, which showed that miR-206 repressed GC cell proliferation, colony formation, invasion, and migration. Finally, the ability of miR-206 to suppress metastasis of SCG-7901 cells into the liver provided a clear explanation for the correlation between low levels of miR-206 and invasive and metastatic gastric cancers. Recently STC2 was identified as a predictive marker for lymph node metastasis in esophageal squamous-cell carcinoma [20]. Expression of TrkB and BDNF is associated with poor prognosis in NSCLC patients[21]. Several studies have reported that IGF1R expression is elevated in metastatic prostate cancer and hormone resistance progression [22,23]. BCL2 expression in primary prostate cancer is a marker for poor prognosis, with an increased risk for recurrence [24]. What is said above suggesting a common oncogenic role in human cancer. Further we identified STC2, HDAC4, KLF4, IGF1R, FRS2, SFRP1, BCL2, BDNF and K-ras as putative functional targets of miR-206, as part of them were talked above. Taken together, our findings suggest a tumor suppressor role of miR-206 in human gastric cancer.

Evidence of miR-206 as a tumor growth suppressor has been reported in several cancers: miR-206 was first found to be downregulated in ERalpha-positive human breast cancer tissues and introduction of miR-206 into estrogen-dependent MCF-7 breast cancer cells inhibits cell growth in a dose- and time-dependent manner [25]. Moreover, miR-206 could promote myogenic differentiation and block tumor growth in xenografted mice by downregulating the product of the MET proto-oncogene: Met tyrosine-kinase receptor [26]. Yan also found that inhibition of miR-206 function could contribute to aberrant cell proliferation and migration, leading to rhabdomyosarcoma development by suppression of C-Met [16]. Meanwhile miR-206 could be used to inhibit HeLa cell migration and focus formation by inhibiting both Notch3 protein and mRNA [27]. Furthermore, miR-206 was downregulated in high metastasis lung cancer compared to low metastasis tumors and normal lung tissues, overexpression of miR-206 inhibited migration and invasion of lung cancer cells [18]. MiR-206 expression decreased in estrogen receptor-α (ERα)-positive endometrial endometrioid adenocarcinoma (EEC) and its overexpression inhibited ERα-dependent proliferation, impaired invasiveness and induced cell cycle arrest of ERα-positive EEC cell lines [28]. All these findings indicated that miR-206 may play a tumor suppressor role in various cancers.

Metastasis contributes to most cancer related deaths. GC ranks as the second leading cause of cancer-related death worldwide because its high rate of metastasis, including lymph node, peritoneum, liver etc. Especially the 5-year survival rate of gastric tumor with liver metastases does not exceed 10% and the median survival without any treatment is about 3 to 5 months [29]. Multiple genes played a pivotal role in GC metastasis: such as the overexpression of insulin-like growth factor receptor-I (IGF-IR) and the activation of its signaling pathways both play critical roles in the development and progression of gastric cancer. Knocking-down of Spl expression by small inhibitory RNA led to decreased IGFIR expression and attenuated growth and metastasis of gastric cancer cells [30], otherwise, IGF1R can be downregulated by miR-7 and significantly reduced GC cell migration and invasion [31]. Adachi found that blockade of IGF-IR is involved in the suppression of cancer cell invasion through downregulation of matrilysin [32]. Those metastasis related genes may provide us new therapeutic targets for GC treatment, as surgical operation can not solve all problems. A surgical resection of liver metastasis from gastric cancer is rarely indicated, because liver metastasis is often associated with extrahepatic disease, such as peritoneal dissemination, lymph node metastasis, and direct cancer invasion of other organs [33]. Recurrent tumors usually develop in the liver following a hepatic resection for gastric metastases (62%–79%) [34]. The efficacy of adjuvant chemotherapy after resection of liver metastases has not been fully evaluated. will assist in the development of new targeted therapies and perhaps best define those patients with potentially chemosensitive tumors. Our findings may have a therapeutic potential to suppress GC metastasis.

## Conclusion

In conclusion, we show, for the first time, that miR-206 is down-regulated in gastric cancer, overexpression of miR-206 decrease the proliferation and metastatic potential of gastric cancer cells in vitro and vivo. Down-regulation of miR-206 was particularly significant in tumors with lymphatic metastasis, local invasion, and advanced TNM staging. We also identified a likely novel mechanism of miR-206 to suppress tumor growth and metastasis by inhibiting the protein translation of STC2, HDAC4, KLF4, IGF1R, FRS2, SFRP1, BCL2, BDNF, and K-ras. Thus, miR-206 functions as a tumor suppressor in gastric cancer. The identification of miR-206 and its target gene in gastric cancer may help in understanding the potential molecular mechanisms of gastric cancer development and may have diagnostic as well as therapeutic value in the future.

## Competing interests

The authors declare that they have no competing interests.

## Authors’ contributions

This project was completed in SYC’s laboratory. LMT and SYC designed and funded the project. JR, HJH, YG, and SY conducted the experiments and analyzed the data. JR and SYC wrote the manuscript. All authors read and approved the final manuscript.

## Supplementary Material

Additional file 1: Table S1 Putative miR-206 recognition sites. Click here for file

Additional file 2: Figure S1 (A) Distribution of miR-1 expression in a cohort of 35 human GC and noncancerous tissues by qRT-PCR. The endogenous U6 RNA was used as the internal control. (B) Pairwise comparison of miR-1 expression between GC and matching non-cancerous tissues showing miR-1 expression was reduced in 54% (19/35) of the sample pairs. (C) Relative expression of miR-1 in four GC cell lines and a normal gastric cell line (GES-1). Click here for file

Additional file 3: Figure S2 (A) Distribution of miR-133a expression in a cohort of 35 human GC and noncancerous tissues by qRT-PCR. The endogenous U6 RNA was used as the internal control. (B) Pairwise comparison of miR-133a expression between GC and matching non-cancerous tissues showing miR-133a expression was reduced in 80% (28/35) of the sample pairs. (C) Relative expression of miR-1 in four GC cell lines and a normal gastric cell line (GES-1). Click here for file
