# Supplementary material for: MicroRNA-206 suppresses gastric cancer cell growth and metastasis
Source: Cell Biosci. 2014 May 5;4:26. doi: 10.1186/2045-3701-4-26 (PMC4030529; doi:10.1186/2045-3701-4-26)
Supplement: Additional file 1: Table S1 — Putative miR-206 recognition sites. [file 2045-3701-4-26-S1.pdf]

Supplementary Table 1 Putative miR-206 recognition sites

GJA1	NM_000165	
	478-485	5' ...UAAGUCCCUGCUAAAACAUUCCA...3'
	1609-1616	5' ..UUACUAAUUUGUUUGACAUUCCA... 3'
BDNF	NM_170731	
	220-226	5' ...UAAAAAGUCUGCAUUACAUUCCU...3'
	390-396	5' ...AAAAUUUGAACCAAAACAUUCCG...3'
	1322-1329	5' ...GGCAUGGUAUUUGAGACAUUCCA...3'
FN1	NM_002026	
	683-690	5' ...UUUAAUAAAAGAUUUACAUUCCA...3'
SFRP1	NM_003012	
	3046-3053	5' ...CUGUGAAAAUGUUUUACAUUCCA...3'
IGF1	NM_153634	
	185-192	5' ...GAAAUACACAAGUAAACAUUCCA...3'
STC2	NM_003714	
	58-65	5' ...UUUUCUUAUCUAUGGACAUUCCA...3'
FRS2	NM_006654	
	3955-3962	5' ...UAAUUUCUUUUAUAAACAUUCCA...3'
TNS3	NM_022748	
	103-110	5' ...AAACUAGUAAACCUGACAUUCCA...3'
HDAC4	NM_006037	
	2334-2340	5' ...UGAAGCCACCAGUUUCAUUCCAA...3'
	3514-3520	5' ...UUCUUUUUGAUCAGAACAUUCCU...3'
	3547-3553	5' ...UCACAGCCACGUGCUCAUUCCAU....3'
ESR1	NM_000125	
	172-178	5' ...GGCUCUACUUCAUCGCAUUCCU......3'
IGF1R	NM_000875	
	4834-4839	5' ...UGGUAUGCCUUGGCCCAUUCCA.....3'
KLF4	NM_004235	
	220-226	5' ...GACUGGAUCUUCUAU--CAUUCCAA.3'
MET	NM_000245	
	499-505	5' ...UCACCCAUUAGGUAAACAUUCCC...3'
	814-820	5'...UAAAUUUUUGUAUAGACAUUCCU...3'
NOTCH3	NM_000435	
	658-664	5' ....UCCCUCACUUCACUGCAUUCCAG...3'
HMGA2	NM_003483	
	2359-2367	5' ...UAAAAAUAAAAGUCGCAUUCCA......3'
BCL2	NM_000633	
	4048-4054	5' ...UUUUUAAAUGUAAAACAUUCCC......3'
