# Supplementary figures and images for: MicroRNA-206 suppresses gastric cancer cell growth and metastasis
Source: Cell Biosci. 2014 May 5;4:26. doi: 10.1186/2045-3701-4-26 (PMC4030529; doi:10.1186/2045-3701-4-26)

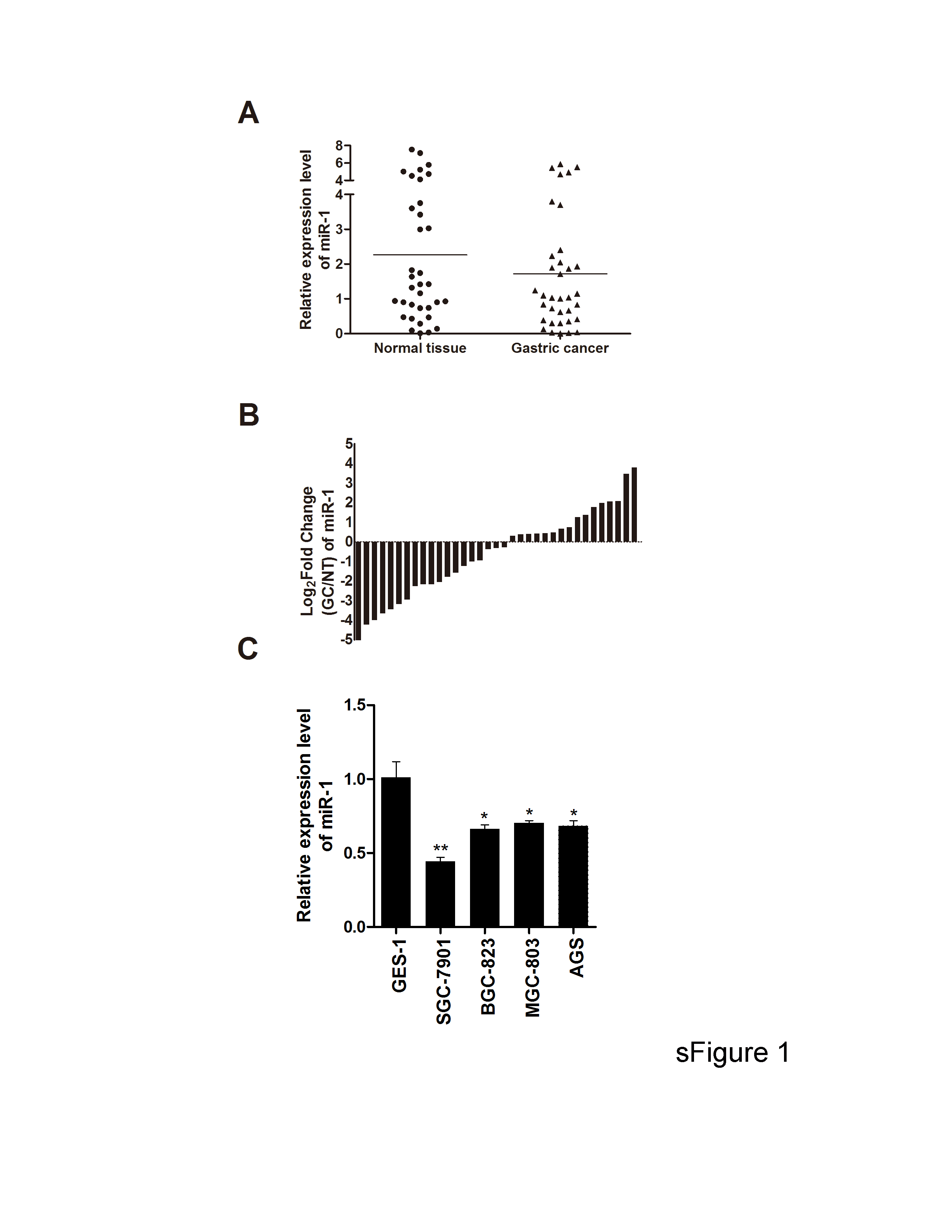

Supplement: Additional file 2: Figure S1 — (A) Distribution of miR-1 expression in a cohort of 35 human GC and noncancerous tissues by qRT-PCR. The endogenous U6 RNA was used as the internal control. (B) Pairwise comparison of miR-1 expression between GC and matching non-cancerous tissues showing miR-1 expression was reduced in 54% (19/35) of the sample pairs. (C) Relative expression of miR-1 in four GC cell lines and a normal gastric cell line (GES-1). [file 2045-3701-4-26-S2.tiff]

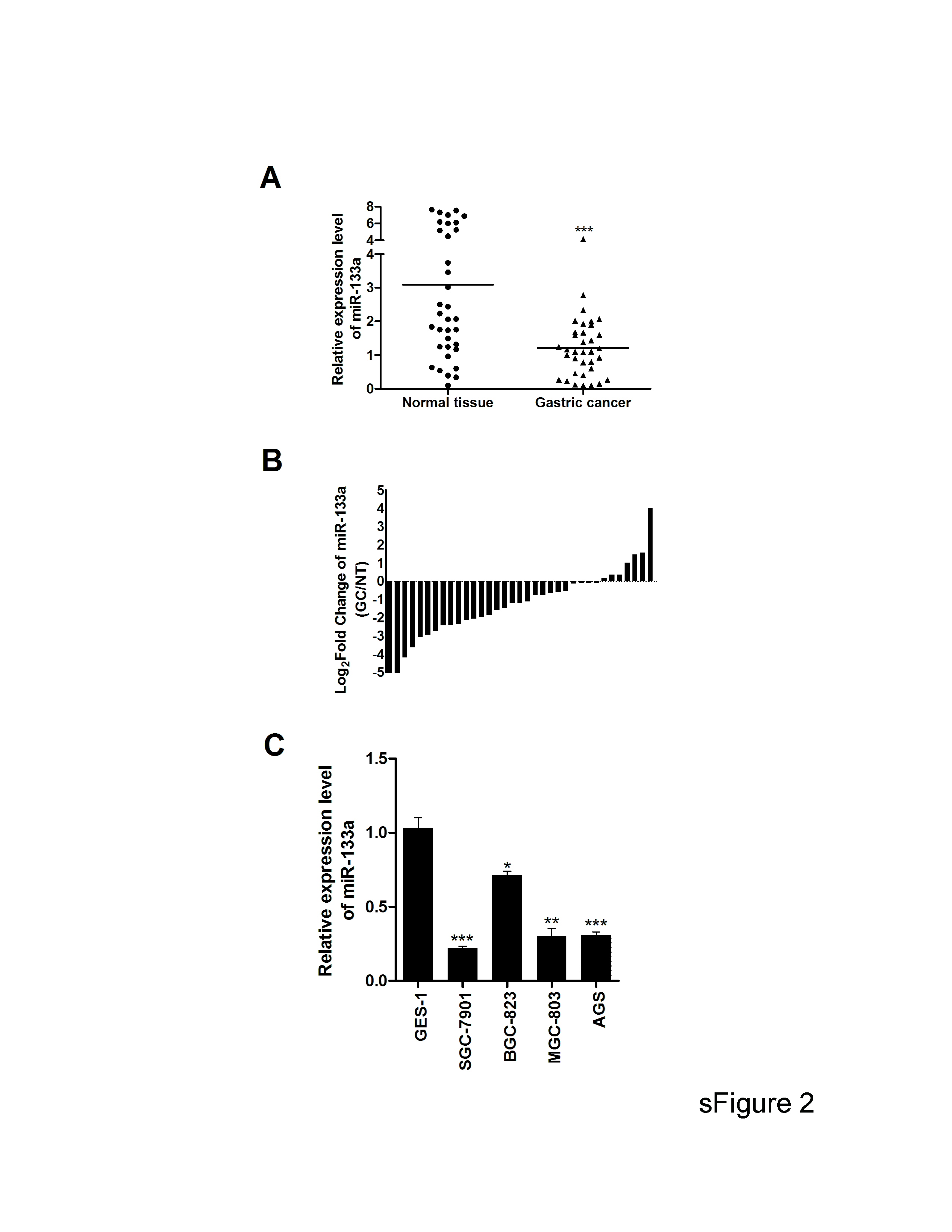

Supplement: Additional file 3: Figure S2 — (A) Distribution of miR-133a expression in a cohort of 35 human GC and noncancerous tissues by qRT-PCR. The endogenous U6 RNA was used as the internal control. (B) Pairwise comparison of miR-133a expression between GC and matching non-cancerous tissues showing miR-133a expression was reduced in 80% (28/35) of the sample pairs. (C) Relative expression of miR-1 in four GC cell lines and a normal gastric cell line (GES-1). [file 2045-3701-4-26-S3.tiff]
